# A prospective, double‐blinded, randomized head‐to‐head clinical trial of topical adapinoid (oleyl adapalenate) versus retinol

**DOI:** 10.1002/ski2.469

**Published:** 2024-11-04

**Authors:** Nhi Nguyen, Nasima Afzal, Mildred Min, Nabeel Ahmad, Laila Afzal, Waqas Burney, Cindy J. Chambers, Raja K. Sivamani

**Affiliations:** ^1^ Integrative Skin Science and Research Sacramento California USA; ^2^ California Northstate University College of Medicine Elk Grove California USA; ^3^ College of Medicine University of Houston Houston Texas USA; ^4^ Pacific Skin Institute Sacramento California USA; ^5^ Department of Dermatology University of California‐Davis Sacramento California USA

## Abstract

**Background:**

Retinoids, such as retinol, are widely investigated and utilized in skin care products as a treatment for photoaging but their use is limited by tolerability. Adapinoid (oleyl adapalenate, OA) is a novel third generation retinoid that is a pro‐drug of adapalene, but there is little research on its effects on photoaging or its tolerability.

**Objectives:**

The purpose of this study is to compare the effects and tolerability of OA 0.5% to retinol 0.5% cream regarding visible signs of facial photoaging including facial wrinkles, fine lines and pigmentation.

**Methods:**

In this 12‐week, double‐blind, randomized clinical trial, 48 eligible participants were recruited and enroled from the Greater Sacramento region. The study consisted of a baseline and follow‐up visits at weeks 4, 8 and 12. Participants were randomized to receive either topical OA 0.5% or retinol 0.5% for 12 weeks. The primary outcome was changes in the appearance of wrinkle severity at 12 weeks. Secondary outcome measures include changes in erythema, skin pigmentation, skin hydration and transepidermal water loss (TEWL).

**Results:**

OA improved wrinkle severity by 9.45% (*p* < 0.0001) at week 12, whereas retinol improved wrinkle severity by 4.11% (*p* < 0.001) compared to baseline. When comparing the two treatment groups at week 12, the OA group improved significantly more than the retinol group (*p* = 0.001). OA decreased pigment intensity at week 12 by 3.88% (*p* < 0.0001), whereas retinol decreased pigment intensity by 3.15% (*p* < 0.03) compared to baseline. OA‐based improvement in pigment intensity at week 12 was not significantly different from retinol (*p* = 0.62). OA reduced facial erythema by 13.39% (*p* < 0.05) at week 12, whereas the retinol group did not have a significant change. OA use led to a 14.92% decrease in TEWL by week 12 (*p* = 0.07), whereas the retinol group had no significant change. OA was better tolerated than retinol when assessed at all follow‐up visits.

**Conclusions:**

OA 0.5% is superior to retinol 0.5% in improving wrinkle severity and similar in improvement of pigment intensity. OA is better tolerated than retinol. Overall, the use of OA as a precursor to adapalene may be an effective method to improving the tolerability of retinoids while maintaining efficacy.

**Trial Registration:**

This study was registered on www.clinicaltrials.gov (NCT05778760).

## Graphical abstract

Adapinoid (oleyl adapalenate), a novel third‐generation retinoid acts as a pro‐drug of adapalene but there is limited research surrounding its efficacy for photoaging. In this 12 week, double‐blinded study, 48 enroled subjects were assessed on the effects and tolerability of topical adapinoid 0.5% versus topical retinol 0.5% on visible signs of photoaging. The clinical study shows that the adapinoid outperformed retinol in the appearance of wrinkle severity, erythema intensity, and tolerability.

**What is already known?**

- Retinol is a widely used retinoid that has been previously shown to improve facial photoaging. However, adapinoid (oleyl adapalenate, OA), a third‐generation retinoid, which is available over‐the‐counter is a precursor to adapalene and has not been clinically studied for its effects on the skin.

**What does this study add?**

- This double‐blind, randomized head‐to‐head study evaluates the topical use of OA compared to retinol for facial photoaging. This study shows that topical application of OA significantly improves the appearance of facial wrinkle severity and erythema intensity and had similar improvement in facial pigment intensity.

**What is the translational message?**

- The use of topical OA is an effective way to improve photoaging and tolerability in comparison to retinol.

## 1 INTRODUCTION

Retinoids have been in high demand due to their versatility in treating various conditions including photoaging. The estimated market value of retinoids is expected to hit 2.5 billion by 2032.^1^ Retinol is widely available for over‐the‐counter use and there are few other next generation retinoids that are available over‐the‐counter. Adapinoid, also known as oleyl adapalenate (OA), is a novel precursor to the third‐generation retinoid adapalene and is available over‐the‐counter. Although OA theoretically has greater lipophilicity and potential to penetrate past the stratum corneum, there are no clinical studies that have evaluated its effects on the skin.

Retinoids are classified into four distinct generations, each characterized by increasing affinity towards the retinoic acid receptor (RAR).^2^ Retinoids bind through direct ligand–receptor binding to induce transcription of retinoic acid‐responsive genes.^3^ Retinoids function to increase epithelial cell turnover and accelerate the shedding of corneocytes by influencing the proliferation and differentiation of cells.^3^ Among the four generations of retinoids, the first and third are commonly used topically in dermatology to treat cutaneous conditions such as photoaging or acne vulgaris.^2^ Manifestation of photoaging as a consequence of cumulative ultraviolet radiation (UVR) includes fine lines, wrinkles and skin pigmentation.^4^ A growing number of studies have demonstrated the role of retinol in improving signs of cutaneous ageing.^5^, ^6^, ^7^, ^8^, ^9^ An open‐label, 10‐week study found significant improvements of fine lines and wrinkles with the use of a 0.5% retinol topical cream in combination with niacinamide, resveratrol and hexylresorcinol.^6^ Another study conducted on an area of sun‐protected skin with 0.4% topical retinol found significant anti‐ageing effects through the epidermal keratinocytes, dermal endothelial cells and fibroblasts.^7^ Furthermore, there has been emerging studies reporting Bakuchiol, a phytochemical deriving from the plant *Psoralea corylfolia*, may serve as an alternative to retinol, demonstrating comparable efficacy in improving photoaging with greater tolerability.^10^, ^11^, ^12^

There are four main forms of retinoids: retinyl esters, retinol, retinaldehyde and retinoic acid. At initial contact with the skin, retinyl esters are hydrolysed to retinol which is then oxidized to retinaldehyde and lastly to retinoic acid.^13^ Subsequently, retinyl esters are the most stable but least potent, whereas retinol and retinaldehyde are more potent in comparison but less stable without other formulation methods to stabilize it.^13^ Retinol is frequently seen in cosmeceutical treatment because it can efficiently penetrate the stratum corneum due to its lipophilic properties.^14^ When retinol interacts with the receptors of the keratinocytes, it can promote cellular proliferation, strengthen the epidermis, reduce transepidermal water loss (TEWL), and prevent collagen degradation.^14^

**FIGURE 1:**
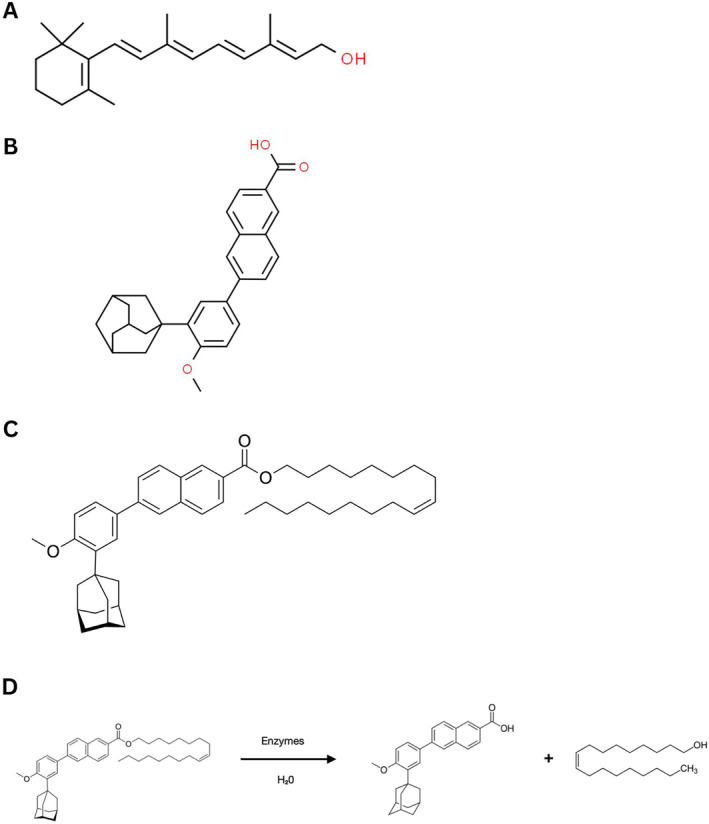
(a) Chemical structure of retinol (ChemSpider ID: 393,012). (b) Chemical structure of adapelene (ChemSpider ID: 54,244). (c) Chemical structure of OA. (d) Visual representation of the pro‐drug and the enzymatic conversion concept from OA to adapalene. OA, oleyl adapalenate.

Retinoids are composed of three fundamental components: a trimethylated cyclohexene ring, a conjugated tetraene side chain and a polar carbon‐oxygen functional group.^15^ Retinol is a 20‐carbon molecule which includes a cyclohexenyl ring, a side chain with four double bonds and an alcohol end group. Adapalene is a third‐generation synthetic retinoid with a naphthoic acid core, a substituted adamant group, and a methoxyphenyl ring attached, it exhibits a higher selectivity for the RAR.^16^ OA is a novel precursor to adapalene and contains an ester group through the addition of oleyl alcohol, which is predicted to undergo enzymatic conversion to transform into adapalene once in the epidermis (Figure 1d) due to the presence of esterases in the epidermis.^17^, ^18^ Ester groups in skin care may function as an emollient assisting in skin moisture and hydration.^19^ Although adapalene has been shown to lead to improvements in photoaging,^20^ no previous clinical studies have evaluated the effects of OA, even though it is available in over‐the‐counter products in the United States.

The aim of this clinical trial is to investigate the effects of the pro‐drug OA (0.5%) on the appearance of photoaging and compare it against the widely used ingredient retinol (0.5%).

## 2 MATERIALS AND METHODS

### 2.1 Subjects

This 12‐week, double‐blind, randomized clinical study was conducted in the Sacramento region at Integrative Skin Science and Research from May 2023 to August 2023. Inclusion criteria included healthy men and women between the ages of 35 and 65 years old. Exclusion criteria included those who had modified their hormonal‐based contraception within three months preceding study entry, current tobacco smokers, individuals with an excess of a 10 pack‐year history of tobacco use, subjects that were pregnant or breastfeeding, prisoners or subjects who were otherwise unable to provide consent. Individuals who had undergone facial cosmetic procedures or treatments within the past 3 months or those who were unwilling to refrain from such procedures during the duration of the study were excluded. Additionally, individuals who had used topical retinoids, acetyl zingerone, vitamin C, bakuchiol, hyaluronic acid or benzoyl peroxide‐containing products within 2 weeks before starting the study or those unwilling to undergo a washout period prior to enrolment were also excluded from participation. This study protocol was approved by the Allendale Institutional Review Board and all subjects provided signed informed consent prior to participation. The study was registered on clinicaltrials.gov (NCT05778760).

### 2.2 Investigational products

Participants were block‐randomized a priori in a 1:1 fashion with an online randomising software (randomizer.org) and the allocation was blinded and performed in sequential fashion by the clinical research coordinators utilising pre‐sealed envelopes to receive either the topical retinol 0.5% (Retinol 10 SU, BASF, Florham, NJ) or topical oleyl adapelenate (Adapinoid, Actera Ingredients Inc, Newton, PA) 0.5% as provided in unmarked tubes from Actera Ingredients. In both formulations, the base water‐based cream vehicle was identical and consisted of pisum sativum peptide, sodium stearoyl lactylate, xanthan gum, trisodium citrate, magnesium stearate, glycerine, caprylyl glyceryl ether, caprylhydroxamic acid, propanediol, citric acid and squalane (5%). Soybean oil was included at 4.5% in the OA formulation to match the soybean oil content from the Retinol 10 SU formulation for retinol. Participants, clinical research coordinators and investigators were all blinded to the assignments. Participants were instructed to apply one to two pumps of the study product once in the evening and spread evenly on a thin film over the entire face. Participants were also given a product log to record daily usage as instructed until their follow‐up visits.

### 2.3 Study visits and procedures

This study comprised a total of five in‐person clinic visits, which included the following: screening and consent, baseline assessment and follow‐up visits at weeks 4, 8 and 12. TEWL and skin hydration were assessed using the VapoMeter (Delfin Technologies, Kuopio, Finland) and the Skin MoistureMeterSC (Delfin Technologies), respectively, at the baseline visit and follow‐up visits at weeks 4, 8 and 12. Facial photographs were captured and analysed through the BTBP 3D Clarity Pro Facial Modelling and Analysis System (Brigh‐Tex BioPhotonics). This image‐based analysis was conducted during the baseline visit and repeated at weeks 4, 8 and 12. Participants at their week 4, 8 and 12 visits received a subjective questionnaire designed as a self‐perception survey for skin assessment and a product tolerability survey.

### 2.4 Statistical analysis

The Shapiro–Wilk test was utilized to assess normality, and comparisons between the two groups were performed using paired, two‐tailed Student's *t*‐tests for baseline comparisons and a two‐tailed Student's *t*‐test for between group comparisons. Statistical significance was set at *p* ≤ 0.05. Results were presented as mean with standard error of the mean. Data were compared within each group against baseline and then between the two treatment groups at each time point. For data visualization Prism v. 10 (GraphPad Software LLC), a statistical graphing software, was used.

## 3 RESULTS

**FIGURE 2:**
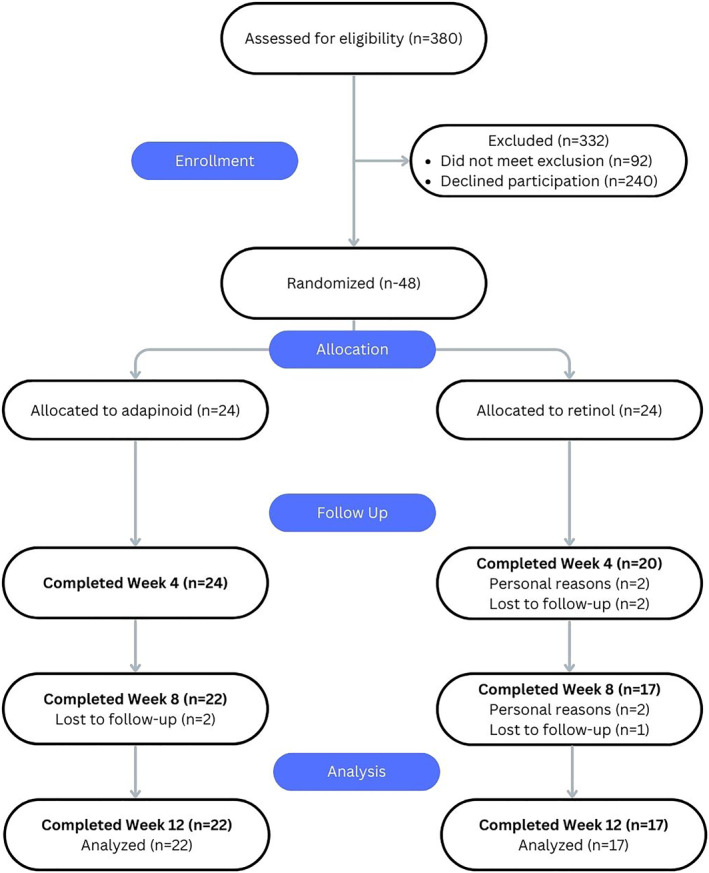
CONSORT flow diagram of enrolment.

A total of 380 people were assessed for eligibility. Of 380 individuals, 240 declined participation, whereas 92 individuals did not meet the inclusion criteria. Among the 48 eligible participants, 48 participants were enroled and 39 participants completed all visits per‐protocol; 7 participants dropped out due to personal reasons and 2 were lost to follow‐up (Figure 2). In total there were, 48 participants enroled with an average age of 51 ± 9 years in the OA group and 53 ± 7 years in the retinol group. One participant in the OA group was a male and the rest of the subjects were female.

### 3.1 Wrinkle severity

**FIGURE 3:**
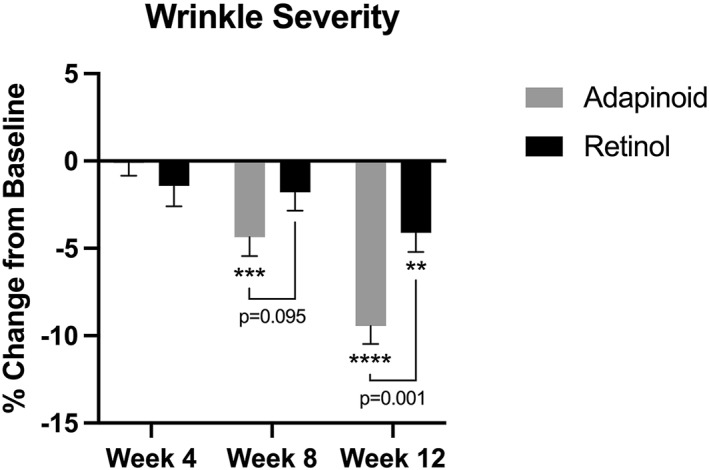
Wrinkle severity was measured using the BTBP 3D clarity Pro facial modelling and analysis system to perform image‐based analysis. Wrinkle severity percent change in the OA group (a) and the retinol group (b) at week 4, week 8 and week 12 compared to their baseline. OA, oleyl adapalenate.

Wrinkle severity is shown in Figure 3. In the OA group, there was no significant change in wrinkle severity at week 4 but by week 8, there was a 4.36% decrease in wrinkle severity (*p* < 0.001) and a 9.45% decrease at week 12 (*p* < 0.0001) when compared to baseline. The retinol group had no significant change in wrinkle severity at week 4, had a 1.80% trending decrease in wrinkle severity at week 8 (*p* = 0.09), and a 4.11% decrease in wrinkle severity at week 12 (*p* < 0.001) compared to baseline. When comparing between the groups, the OA group had trend for greater reduction at week 8 (*p* = 0.095) and greater reduction in facial wrinkle severity at week 12 (*p* = 0.001).

### 3.2 Pigment intensity

**FIGURE 4:**
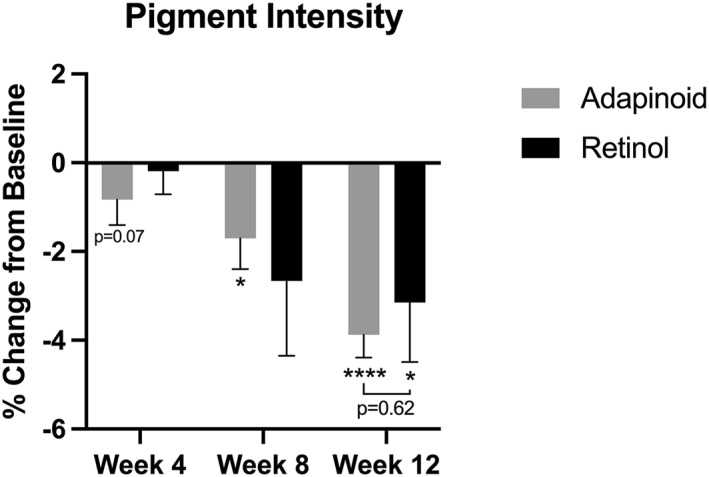
Pigment intensity was measured using the BTBP 3D Clarity Pro Facial Modelling and Analysis System to perform image‐based analysis. Pigment intensity percent change in the aleyl adapalenate group (a) and the retinol group (b) at week 4, week 8 and week 12 compared to their baseline.

Pigment intensity is shown in Figure 4. The OA group had a 1.70% decrease at week 8 (*p* < 0.025) and 3.88% decrease by week 12 (*p* < 0.0001) in pigment intensity compared to baseline. The retinol group had no significant change in pigment intensity at week 4 or week 8 and exhibited a significant 3.15% decrease in pigment intensity at week 12 (*p* < 0.03) when compared to baseline. There were no significant changes in pigment intensity between the OA and the retinol treatment groups at week 4, 8 or 12.

### 3.3 Erythema intensity

**FIGURE 5:**
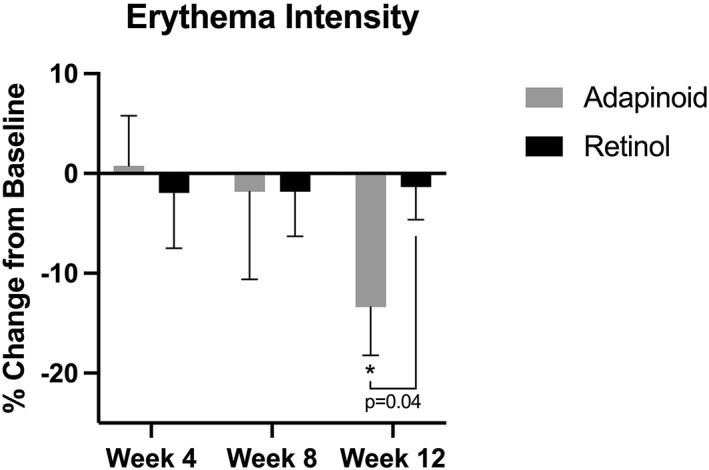
Erythema was measured using the BTBP 3D Clarity Pro Facial Modelling and Analysis System to perform image‐based analysis. (a) Erythema percentage change in the OA group at week 4, week 8 and week 12 compared to baseline. (b) Erythema percentage change in the retinol group at week 4, week 8 and week 12 compared to baseline. OA, oleyl adapalenate.

Erythema intensity is shown in Figure 5. The OA group did not have significant change at weeks 4 or 8, but there was a 13.39% decrease in erythema intensity at week 12 (*p* < 0.05) when compared to baseline. The retinol group did not have a significant change in erythema intensity at week 4, 8 or 12 compared to baseline. Treatment with OA significantly reduced facial erythema compared to the retinol group at week 12 (*p* < 0.05).

### 3.4 Skin transepidermal water loss (TEWL)

**FIGURE 6:**
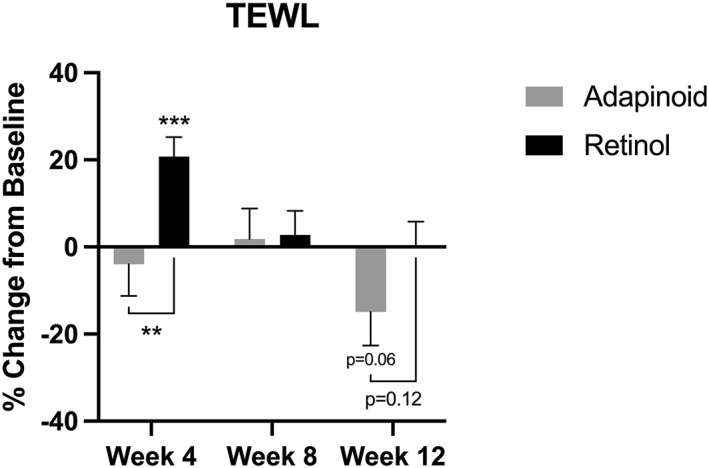
TEWL percent change in the OA group (a) and retinol group (b) at week 4, week 8 week 12 compared to their baseline. OA, oleyl adapalenate.

TEWL is shown in Figure 6. In the OA group, there was no significant change at weeks 4 or 8 and a 14.9% decrease by week 12 (*p* = 0.07) when compared to baseline. In the retinol group, there was a significant 20.7% increase in TEWL at week 4 (*p* = 0.0002) and no significant changes at weeks 8 and 12 compared to baseline. There was a significant difference in TEWL between the OA and retinol groups at week 4 (*p* = 0.006); however, the differences between groups at week 12 were not significant (*p* = 0.12).

### 3.5 Skin hydration

**FIGURE 7:**
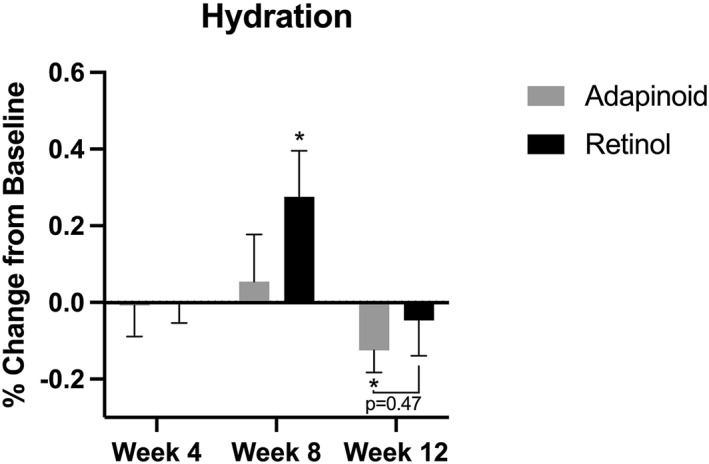
Skin hydration percent change in the OA group (a) and the retinol group (b) at week 4, week 8 and week 12 compared to their baseline. OA, oleyl adapalenate.

Skin hydration is shown in Figure 7. The OA group had no significant change in skin hydration at weeks 4 and 8 when compared to baseline. At week 12, there was a significant 0.12% decrease in skin hydration (*p* = 0.043) when compared to baseline. The retinol group did not have any significant changes in skin hydration at week 4 or 12 when compared to baseline. At week 8, there was a significant 0.27% increase in skin hydration (*p* = 0.033) when compared to baseline. There were no significant differences in skin hydration between the two cohorts at week 4, 8 or 12.

### 3.6 Tolerability

Subjective ratings of tolerability are shown in Table 1. The 3‐point scale referred to ‘0’ as none, ‘1’ as mild, ‘2’ as moderate and ‘3’ as severe. Overall, the OA group reported less tolerability‐related side effects compared to the retinol group at weeks 4, 8 and 12. In particular, at week 12, 5% reported burning and 10% reported scaling in the OA group compared to 11% reporting itching, 21% reporting burning, 16% reporting stinging, 21% reporting scaling and 21% reporting redness in the retinol group as shown in Table 2.

**TABLE 1:** Demographic data.

	Oleyl adapalenate	Retinol	Overall
Average age	50.5	53.4	53.1
Standard deviation	9.09	7.45	8.16
Male	1	0	1
Female	22	25	47
Skin type I	10	9	19
Skin type II	3	5	8
Skin type III	4	5	9
Skin type IV	2	4	6
Skin type V	1	2	3
Skin type VI	2	1	3

**TABLE 2:** Tolerability assessment responses.

	Oleyl adapalenate group				Retinol group			
	None 0	Mild 1	Moderate 2	Severe 3	None 0	Mild 1	Moderate 2	Severe 3
Week 4								
Itching	91%	9%	0%	0%	82%	14%	5%	0%
Burning	86%	14%	0%	0%	55%	32%	9%	5%
Stinging	91%	9%	0%	0%	68%	23%	5%	5%
Scaling	82%	18%	0%	0%	59%	36%	5%	0%
Redness	95%	5%	0%	0%	55%	32%	9%	5%
Week 8								
Itching	100%	0%	0%	0%	84%	16%	0%	0%
Burning	100%	0%	0%	0%	84%	16%	0%	0%
Stinging	100%	0%	0%	0%	89%	11%	0%	0%
Scaling	80%	20%	0%	0%	84%	16%	0%	0%
Redness	95%	5%	0%	0%	74%	26%	0%	0%
Week 12								
Itching	100%	0%	0%	0%	89%	11%	0%	0%
Burning	95%	5%	0%	0%	79%	21%	0%	0%
Stinging	100%	0%	0%	0%	84%	16%	0%	0%
Scaling	90%	10%	0%	0%	79%	21%	0%	0%
Redness	100%	0%	0%	0%	74%	21%	5%	0%

**FIGURE 8:**
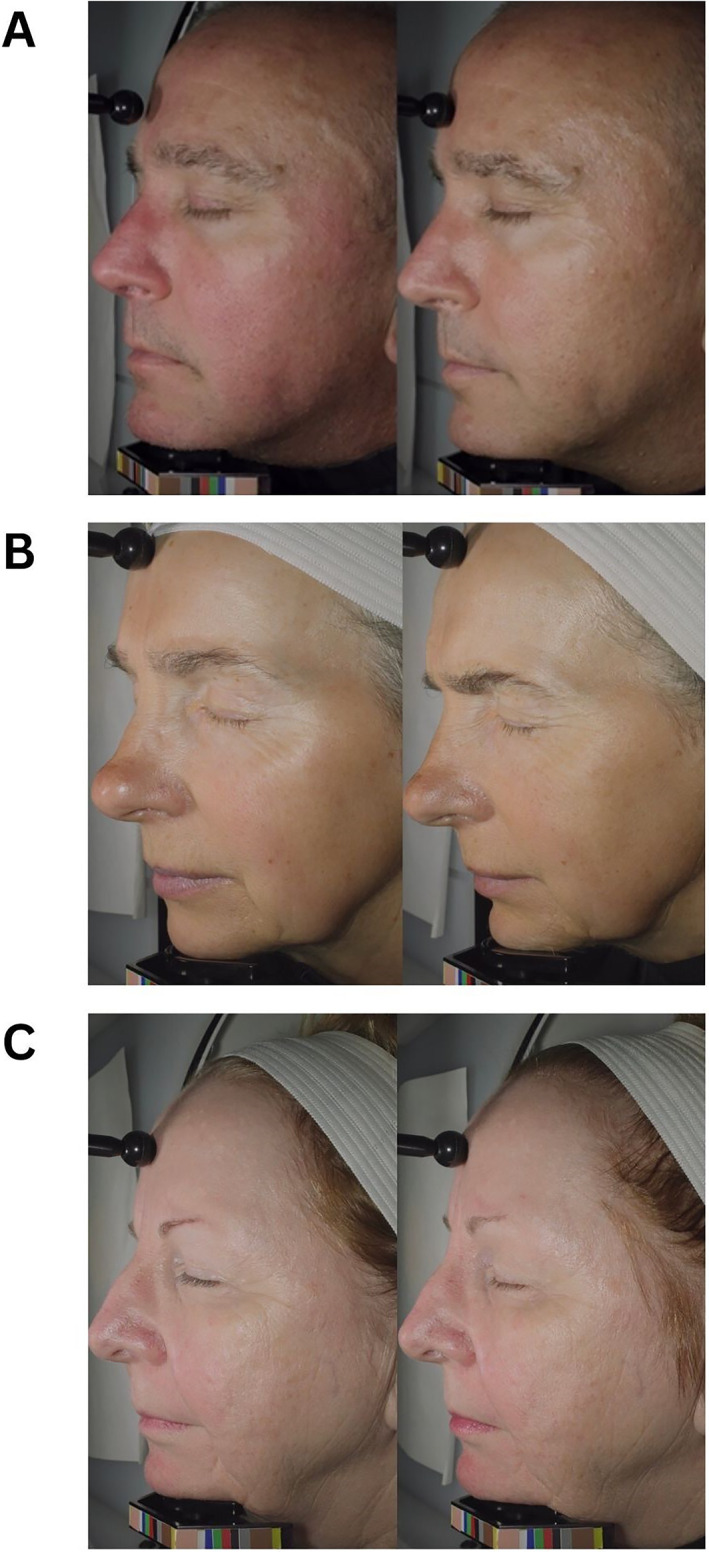
(a) From left to right, OA subjects at baseline and week 12. (b) From left to right, OA subjects at baseline and week 12. (c) From left to right, retinol subjects at baseline and week 12. OA, oleyl adapalenate.

Representative photos of the subjects are shown in Figure 8.

### 3.7 Adverse events

There were no adverse reactions reported for the duration of the study.

## 4 DISCUSSION

The findings from our double‐blinded, randomized clinical trial demonstrate that a 12‐week regimen of daily oleyl adapalenate application resulted in a reduction in wrinkles, facial erythema, pigment intensity and TEWL. OA performed just as effectively as retinol in pigment intensity reduction and outperformed retinol in reduction of both wrinkle severity and erythema. Furthermore, OA was much better tolerated at all weeks of assessment. Although there was previous theoretical potential for OA as a pro‐drug to adapalene, this study demonstrates that topical OA has clinical photoaging benefits that are typical for retinoids but with greater efficacy and tolerability compared to retinol.

There was differential effect on skin TEWL between the OA and retinol treatments. Subjects in the retinol group displayed an increase in TEWL after 12 weeks of use. This is in agreement with previous clinical assessment of retinol showing an 88% increase in TEWL.^21^ On the other hand, the OA group had no change in the TEWL at weeks 4 and 8 and a decrease at week 12. This is in agreement with previous adapalene‐based studies that showed that exposure to topical 0.1% adapalene did not result in any change in the TEWL.^22^

The retinol group had a significant reduction in wrinkle severity and this finding is consistent with other studies after at least 12 weeks of application. This effect may be attributed to retinol's upregulation of COL1A1 and COL3A1 gene expression, correlating with increased collagen synthesis,^23^, ^24^ a pivotal contributor to wrinkle reduction which our imaging data supports. OA has superiority over retinol in reducing wrinkle severity may be in part due to its chemical structure. The differences in lipophilicity between retinol, OA and adapalene are evidenced by their chemical structure with OA having an ester group (Figure 1c). The added ester group promotes lipophilicity of OA and thus may allow it to penetrate more quickly and deeper into the stratum corneum.^25^ This is reflected by OA's greater octanol: water partition coefficient at 10.47 compared to adapalene at 6.06, making adapinoid more lipophilic. OA's greater partition coefficient may allow it to permeate the stratum corneum more effectively than adapalene. It is reported that the highest enzyme activity of esterases occur in the epidermis^17^, ^18^ and may allow the more lipophilic OA to penetrate and then become adapalene, allowing for a significant reduction in wrinkle severity compared to retinol.^26^

OA was better tolerated than retinol with subjects reporting less itching, burning, stinging, scaling and redness. This is in agreement with previous research that has shown that adapalene (a derivative of OA) is less irritating compared to other topical retinoids.^27^ The enhanced tolerability of OA may be due to its selective affinity to retinoic acid receptors, notably beta and gamma receptors.^2^ The decreased skin irritation reported by the subjects in this study may generally lead to greater compliance compared to retinol.

Although this study was focused on photoaging, the role of retinoids is more broad and may include treatments of inflammatory conditions as well such as psoriasis or acne.^28^ Since our study showed a similar retinoid‐like clinical effect in photoaging, future studies should consider studies in inflammatory conditions such as psoriasis or acne.

### 4.1 Limitations

Our study results represent the clinical results from a single site and future studies should expand the population tested. However, the strength of this study is the double‐blind, comparative, randomized design. Our study results do not inform on longer courses of use since the study was limited to a 12‐week duration and a longer duration of study would be warranted for 6 months to a year. The participants in the study predominantly have Fitzpatrick skin type of I–III and future studies with a more broad recruitment of skin types would allow for greater generalisability of the findings.

## 5 CONCLUSIONS

Our clinical trial demonstrates that oleyl adapalenate outperformed retinol in reducing image analysis‐based facial wrinkle severity and erythema and was similar to retinol for the improvement of image‐based pigment intensity. OA was better tolerated after a 12‐week topical regimen. These findings have important implications for the skincare industry, suggesting that OA may offer a more effective solution for addressing common cosmetic and photoaging concerns with a novel and emerging over‐the‐counter ingredient. The promising results of this study pave the way for future investigations which may ultimately lead to better understanding of how OA can be utilized in the skincare and dermatology space.

## CONFLICT OF INTEREST STATEMENT

RKS serves as a scientific advisor for LearnHealth, Codex Labs and Arbonne and as a consultant to Burt's Bees, Novozymes, Nutrafol, Abbvie, Leo, Almirall, Galderma, Lilly, UCB, Incyte, Sanofi, Novartis, Sun and Regeneron Pharmaceuticals. The other authors report no conflicts of interest.

## AUTHOR CONTRIBUTIONS

**Nhi Nguyen**: Data curation (equal); investigation (equal); writing—original draft (equal). **Nasima Afzal**: Data curation (equal); formal analysis (equal); project administration (equal); writing—original draft (equal). **Mildred Min**: Formal analysis (equal); writing—original draft (equal). **Nabeel Ahmad**: Writing—original draft (equal). **Laila Afzal**: Writing—original draft (equal). **Waqas Burney**: Writing—review and editing (equal). **Cindy J. Chambers**: Writing—review and editing (equal). **Raja K. Sivamani**: Conceptualisation (lead); data curation (equal); formal analysis (equal); funding acquisition (lead); investigation (lead); methodology (lead); supervision (lead); writing—review and editing (lead).

## ETHICS STATEMENT

This study was approved by the Allendale IRB. (Protocol # ADAP_RET).

## PATIENT CONSENT

All participants provided written informed consent prior to participation. The patients also gave consent for publication.

## Data Availability

The data are not publicly available. The data presented in this study are available on request from the corresponding author.
